# Patterns of tobacco use, quit attempts, readiness to quit and self-efficacy among smokers with anxiety or depression: Findings among six countries of the EUREST-PLUS ITC Europe Surveys

**DOI:** 10.18332/tid/98965

**Published:** 2019-02-05

**Authors:** Ioanna Petroulia, Christina N. Kyriakos, Sophia Papadakis, Chara Tzavara, Filippos T. Filippidis, Charis Girvalaki, Theodosia Peleki, Paraskevi Katsaounou, Ann McNeill, Ute Mons, Esteve Fernández, Tibor Demjén, Antigona C. Trofor, Aleksandra Herbeć, Witold A. Zatoński, Yannis Tountas, Geoffrey T. Fong, Constantine I. Vardavas, on behalf of the EUREST-PLUS consortium

**Affiliations:** 1 National and Kapodistrian University of Athens (UoA), Athens, Greece; 2 European Network for Smoking and Tobacco Prevention (ENSP), Brussels, Belgium; 3 University of Crete (UoC), Heraklion, Greece; 4 Division of Prevention and Rehabilitation, University of Ottawa Heart Institute, Ottawa, Canada; 5 Department of Primary Care and Public Health, School of Public Health, Imperial College London, London, United Kingdom; 6 King’s College London (KCL), London, United Kingdom; 7 Cancer Prevention Unit and WHO Collaborating Centre for Tobacco Control, German Cancer Research Center (DKFZ), Heidelberg, Germany; 8 Tobacco Control Unit, Catalan Institute of Oncology (ICO), and Cancer Control and Prevention Group, Bellvitge Biomedical Research Institute (IDIBELL), Catalonia, Spain; 9 School of Medicine and Health Sciences, Universitat de Barcelona, Catalonia, Spain; 10 Smoking or Health Hungarian Foundation (SHHF), Budapest, Hungary; 11 Universitatea de Medicina si Farmacie ‘Grigore T. Popa’ Iași, Iași, România; 12 Aer Pur Romania, Bucharest, Romania; 13 Health Promotion Foundation, Warsaw, Poland; 14 European Observatory of Health Inequalities, President Stanisław Wojciechowski State University of Applied Sciences, Kalisz, Poland; 15 Department of Psychology & School of Public Health and Health Systems, University of Waterloo (UW), Waterloo, Canada; 16 Ontario Institute for Cancer Research, Toronto, Canada

**Keywords:** depression, smoking cessation, anxiety, mental health, Europe

## Abstract

**INTRODUCTION:**

We compared smoking behaviors, past quit attempts, readiness to quit and beliefs about quitting among current cigarette smokers with probable anxiety or depression (PAD) to those without PAD, from six European Union (EU) Member States (MS).

**METHODS:**

A nationally representative cross-sectional sample of 6011 adult cigarette smokers from six EU MS (Germany, Greece, Hungary, Poland, Romania, Spain) was randomly selected through a multistage cluster sampling design in 2016. Respondents were classified as having PAD based on self-reported current diagnosis or treatment for anxiety or depression, or a positive screen for major depression, according to a validated two-item instrument. Sociodemographic characteristics, patterns of tobacco use, past quitting, readiness to quit, self-efficacy and beliefs about quitting were assessed for patients with and without PAD. Logistic regression was used to examine predictors of PAD. All analyses were conducted using the complex samples package of SPSS.

**RESULTS:**

Among smokers sampled, 21.0% (95% CI: 19.3–22.9) were identified as having PAD. Logistic regression analyses controlling for socioeconomic variables and cigarettes smoked per day found smokers with PAD were more likely to have made an attempt to quit smoking in the past (AOR=1.48; 95% CI: 1.25–1.74), made a quit attempt in the last 12 months (AOR=1.75; 95% CI: 1.45–2.11), and report lower self-efficacy with quitting (AOR=1.83; 95% CI: 1.44–2.32) compared to smokers without PAD. Additionally, it was found that individuals with PAD were more likely to report having received advice to quit from a doctor or health professional and having used quitline support as part of their last quit attempt.

**CONCLUSIONS:**

Smokers with PAD report a greater interest in quitting in the future and more frequent failed quit attempts than smokers without PAD; however, the high rates of untreated anxiety or depression, nicotine dependence, low confidence in the ability to quit, infrequent use of cessation methods, as well as socioeconomic factors may make quitting difficult.

## INTRODUCTION

Smoking-related mortality is significantly higher in people with serious mental illness^1^. It is estimated that half of all deaths among individuals with mental illness are attributable to tobacco use^2^. While significant progress has been made in reducing tobacco use within the general population, rates of tobacco use remain high among individuals with mental illness, including individuals with depression and/or anxiety compared with those without such disorders^3,4^. People with depression are about twice as likely to be smokers than individuals who are not depressed^5^.

Individuals with mental illness have been also shown to have greater daily tobacco consumption, nicotine dependence and smoking relapse^4-12^. Smoking cessation often requires numerous attempts by these people and may be accompanied by anxiety and depression during the quit attempt, due in part to nicotine withdrawal symptoms^7^. Thus, smokers with mental health illness may find it more difficult quitting^7^, although highly motivated to quit^13^. Available quitting aids are both safe and effective in supporting cessation in tobacco users with mental illness and stopping smoking is associated with an improvement in mental health rather than deterioration^1,5,14-18^.

The majority of studies published in this area are from large surveys in North America, the United Kingdom and Australia^3,4,7-12,19-21^. There are limited data available to characterize tobacco users in European Union (EU) Member States (MS) with regards to mental disorders, such as anxiety and depression. Given the importance of addressing tobacco use among this key population of tobacco users, gaining a better understanding of similarities and differences between tobacco users with and without mental illness can be critical in informing both health policy and practice.

The current study explores the associations between probable anxiety or depression and smoking behaviors, past quit attempts, readiness to quit, self-efficacy with quitting, as well as beliefs about quitting among adult tobacco users in six EU MS.

## METHODS

### Design and sample

This study was undertaken as part of the European Commission Horizon-2020 study entitled ‘European Regulatory Science on Tobacco: Policy implementation to reduce lung diseases (EUREST-PLUS-HCO-06-2015)’, which aims to evaluate the impact of the European Union (EU) Tobacco Products Directive (TPD)^22^ and the World Health Organization (WHO) Framework Convention on Tobacco Control (FCTC) at European level^23^.

Using the methodology of the International Tobacco Control (ITC) Project, a nationally representative cohort sample of adult smokers from six EU MS (Germany, Greece, Hungary, Poland, Romania, Spain) (ITC 6E) was created using multistage cluster sampling^23,24^. Baseline survey data from this pre- versus post-TPD study design of EUREST-PLUS was collected from a sample of 6011 smokers, age 18 years or older, between June and September 2016, just prior to the implementation of the TPD. Participants were recruited from households using a random walk design from sampled geographical clusters based on the Nomenclature of Territorial Units for Statistics^24^. Approximately 1000 participants from 100 area clusters were sampled in each country, with the aim of obtaining 10 adult smokers per cluster. Clusters were allocated to strata proportionally to population size for age 18 and older. Within each cluster, household addresses were sampled using a random walk design. Face-to-face survey interviews were completed using tablets (CAPI) with one male and one female smoker randomly selected from each household. Respondents were classified as smoker if they had smoked at least 100 cigarettes in their lifetime and were currently smoking cigarettes at least monthly (i.e. daily and non-daily smokers). Screening of households continued until the required number of smokers from the cluster had been interviewed. Further details on the survey methodology are described elsewhere^24^. According to the most recent Eurobarometer, the smoking prevalences for the six individual countries that participated in the project in 2017 were: DE: 25%, GR: 37%, HU: 27%, PL: 30%, RO: 28%, ES: 28% (average: 29%)^25^.

### Measures

#### Sociodemographics

We examined sociodemographic characteristics, including age, sex, and educational level categorized as low (primary, lower and middle [both pre-vocational secondary]), moderate (secondary vocational, senior general secondary and pre-university), and high (higher professional and university Bachelor, university Master). Further, monthly household income was reported in the local currency: Euro (€) for Germany, Greece and Spain, Złoty (zł) for Poland, Forint for Hungary and Leu for Romania, with income categorized as low (<€1750, ≤150000 Ft, ≤2000 zł and ≤1000 lei), moderate (€1750 to €3000, 150001 Ft to 250000 Ft, 2001 zł to 4000 zł, 1001 lei to 2500 lei) and high (>€3000, >250000 Ft, >4000 zł, >2500 lei). Lastly, difficulties paying important bills on time during the last 30 days were assessed with the question: ‘In the last 30 days, because of a shortage of money, were you unable to pay any important bills on time, such as electricity, telephone or rent bills? (yes/no)’.

#### Probable anxiety or depression

Three questions were used to assess the presence of probable anxiety or depression (PAD): 1) a positive screen for major depression (PSD) in past 30 days assessed using a validated two-item instrument, ‘during the last 30 days, have you often been bothered by little interest or pleasure in doing things?’ (yes/no) and ‘during the last 30 days, have you often been bothered by feeling down, depressed, or hopeless?’ (yes/no)^26^. A ‘yes’ response to one or both of these questions was considered a positive screen. This two-item instrument has been shown to have good sensitivity of 95% (95% CI: 88–97%) and a specificity of 65% (95% CI: 55–74%) for detecting major depression^27^; 2) current treatment or diagnosis of depression (TDD) using the question ‘are you currently being treated for, or do you have a current diagnosis for depression?’ (yes/no); or 3) current treatment or diagnosis of anxiety (TDA) using the question ‘are you currently being treated for, or do you have a current diagnosis for anxiety?’ (yes/no). Respondents who replied positively to the questions for PSD, TDD, TDA were referred to as having probable anxiety/depression (PAD).

#### Smoking behaviors

Cigarette smoking status at time of recruitment was assessed with the question: ‘On average, how often do you currently smoke factory-made or roll-yourown cigarettes?’ (response: daily; less than daily, but at least once a week; less than weekly, but at least once a month; and less than monthly). Current cigarette use was defined as using cigarettes daily or less than daily, but at least once a week. Nicotine dependence was assessed by two measures: 1) cigarette consumption (‘On average, how many factory-made or roll-your-own cigarettes do you smoke each day?’), and 2) time to first cigarette (‘On days that you smoke, how soon after waking do you usually have your first smoke?’ with response: >30 minutes, 6–30 minutes, ≤5 minutes)^28^.

#### E-cigarette use

Ever use of electronic cigarettes (e-cigarette), was assessed by the question: ‘Have you ever used an e-cigarette or vaping device, even one time?’ (yes/no/don’t know/refused). Current e-cigarette use was assessed based on responses to the following question: ‘on average, how often, do you currently use e-cigarette or vaping devices?’ (never; daily; less than daily, but at least once a week; less than weekly, but at least once a monthly; and less than monthly). Those who reported using e-cigarettes ‘daily’ or ‘less than daily, but at least once a week’ were defined as current users.

#### Quit attempts and cessation assistance used

Data on quit attempts were collected from respondents with the question: ‘Have you ever tried to quit smoking? (yes/no)’. Respondents who had ever tried to quit smoking were subsequently asked the questions: ‘have you made an attempt to quit smoking in the last 12 months?’ (yes/no); ‘how many quit attempts have you made in the last 12 months?’ (no attempt; 1 attempt; 2 or more attempts); ‘how long ago did your most recent quit attempt start?’ (<1 month ago, 1–3 months ago, 4–6 months ago, ≥7 months ago); and ‘how long did you stay smoke-free on your most recent quit attempt?’ (less than 1 day, 1–6 days, 1–4 weeks, 1–6 months, 7–12 months).

Additionally, respondents who answered ‘yes’ to having ever tried to quit smoking were asked: ‘Which of the following products and services did you use as part of your last quit attempt?’, with yes/no responses for the following quitting methods: 1) nicotine replacement therapy (NRT), such as patches, gum, mouth spray, etc.; 2) other pharmacotherapy, including varenicline (Chantix or Champix), bupropion (Zyban, or Wellbutrin), cytisine (Desmoxan or Tabex); 3) local stop-smoking services such as clinics, specialists, individual or group counselling, stop-smoking courses, or behavior therapy; 4) face-to-face advice from doctor or other health care professional; 5) telephone/quitline services; 6) e-cigarette or vaping device; and 7) other.

#### Self-efficacy and readiness to quit smoking

Self-efficacy with quitting was evaluated with the questions: ‘if you decided to give up smoking completely in the next 6 months, how sure are you that you would succeed?’ (not at all sure; slightly sure; moderately sure; very sure; extremely sure). Perceived difficulty with quitting was assessed using the question: ‘how difficult would it be for you to quit smoking if you wanted to?’ (not at all difficult; slightly difficult; moderately difficult; very difficult; extremely difficult). Readiness to quit was assessed with the question: ‘are you planning to quit smoking?’ (within the next month; within the next 6 months; sometime in the future beyond 6 months; not planning to quit). Respondents who indicated that they plan to quit within the next month, 6 months or in the future beyond 6 months, were also asked: ‘How much do you want to quit smoking?’ (a little, somewhat, a lot).

### Analysis

For all analyses, we applied sample weights for sex and age to yield nationally representative data and analyzed using the complex samples package of SPSS (version 21.0). Missing data were excluded on a case-by-case basis. Chi-squared tests of association were performed to examine the relationship between demographic variables for the total sample, and respondents with and without each of the PAD sub-groups (i.e. positive screen for depression in the past 30 days, diagnosis/treatment of depression, diagnosis/treatment anxiety). We repeated chi-squared analyses to examine the relationship between PAD and the three PAD sub-groups and smoking behaviors, quit attempts, use of quitting methods, quitting intentions and beliefs. Independent samples t-tests were used for the comparison of continuous variables (e.g. age, cigarettes per day) between the two groups. Logistic regression analysis was used to examine factors associated with the presence of PAD, adjusting for sex, age, education, country, ability to pay bills on time and cigarettes smoked per day. All independent variables were recoded as dichotomous variables. Results of the logistic regression analyses are presented as adjusted odds ratios (AOR) and 95% confidence interval (95% CI). All p-values reported are two-tailed. All statistical tests and confidence intervals were corrected for the complex sample design with sampling region as strata and primary sampling unit as clusters.

## RESULTS

### Probable anxiety or depression

Twenty-one per cent (21.0%; 95% CI: 19.3–22.9) of EUREST-PLUS Wave 1 respondents reported PAD in the last month (Table 1). The proportion of smokers with PAD was highest in Romania (26.7%; 95% CI: 22.3–31.6) and lowest in Hungary (11.7%; 95% CI: 9.1–15.1). Across the sample, 18.3% of respondents had a positive screen for depression (PSD) in the last 30 days and 3.6% of respondents reported currently being treated or diagnosed with depression (PDD), while 4.7% reported currently being treated for or diagnosed with anxiety (PDA).

**Table 1:** Prevalence of probable anxiety or depression overall and by country

*Variable*	*Weighted % (95% CI)*						
	*Overall N=5859*	*Germany N=985*	*Greece N=994*	*Hungary N=967*	*Poland N=927*	*Romania N=988*	*Spain N=998*
Probable anxiety or depression (PAD)^1^	21.0 (19.3–22.9)	18.4 (15.2–22.2)	24.0 (19.3–29.4)	11.7 (9.1–15.1)	23.2 (18.8–28.3)	26.7 (22.3–31.6)	22.1 (17.3–27.8)
Anxiety - current treatment/diagnosis (TDA)^2^	4.7 (3.9–5.6)	3.0 (1.9–4.7)	8.2 (5.7–11.6)	4.4 (3.0–6.4)	3.1 (1.7–5.8)	2.3 (1.4–3.9)	7.1 (4.8–10.3)
Depression -current treatment/diagnosis (TDD)^3^	3.6 (2.9–4.5)	5.1 (3.5–7.4)	1.9 (1.0–3.5)	4.4 (2.5–7.7)	3.2 (1.7–6.0)	2.6 (1.7–4.2)	4.3 (2.4–7.8)
Depression - positive screen (PSD)^4^	18.3 (16.6–20.1)	16.3 (13.3–19.8)	20.7 (16.4–25.7)	7.4 (5.7–9.4)	20.4 (16.4–25.1)	26.2 (21.8–31.1)	18.7 (14.0–24.6)

1 PAD = TDA or TDD or PSD.

2 ‘Are you currently being treated for, or do you have a current diagnosis for anxiety?’.

3 ‘Are you currently being treated for, or do you have a current diagnosis for depression?’.

4 Positive screen is defined as a positive response to either of the two items: ‘During the last 30 days, have you often been bothered by little interest or pleasure in doing things?’ or ‘During the last 30 days, have you often been bothered by feeling down, depressed, or hopeless?’.

CI: Confidence Interval. Note: Subjects with full data for the questions concerning Anxiety - current treatment/diagnosis, Depression - current treatment/diagnosis and Depression - positive screen were analyzed (N=5859).

### Sociodemographic characteristics

Sociodemographic characteristics of the sample are presented in Table 2. The bivariate analysis found that smokers with PAD, PSD, TDD and TDA were significantly more likely to be female compared to those without these disorders. PAD, TDA or PSD was positively associated with having a low monthly household income and being unable to pay bills on time, compared to respondents without PAD, TDD or TDA, and were associated with having a lower level of education.

**Table 2:** Characteristics of respondents by presence of probable anxiety or depression, pooled sample

*Variable*	*No PAD (n=4575)*	*Probable Anxiety or Depression (PAD) Definitions*								*Total sample*

		*PAD (n=1284)*		*Current treatment/diagnosis for depression (TDD) (n=224)*		*Current treatment/diagnosis for anxiety (TDA) (n=303)*		*Positive screen for depression (PSD) (n=1115)*		*(n=5859)*

			*p*		*p*		*p*		*p*	
**Male, %**	59.4	50.0	***	42.8	***	47.6	***	49.7	***	57.4
**Age, mean (SE)**	43.3 (0.3)	45.0 (0.6)	ns	46.6 (1.5)	ns	45.4 (0.9)	ns	44.9 (0.6)	ns	43.7 (0.3)
**Highest level of education,** %			ns		***		**		ns	
Low	37.4	38.5		58.7		51.7		37.8		37.6
Moderate	51.4	51.3		38.3		41.4		51.5		51.4
High	11.2	10.2		2.9		6.9		10.6		10.9
**Monthly household income, %**			***		ns		****		***	
Low	24.8	34.8		45.8		40.6		35.8		27.3
Moderate	49.3	47.3		46.7		48.7		46.0		48.7
High	25.9	17.9		7.6		10.7		18.2		24.0
**Unable to pay bills on time, %**	9.6	24.4	***	28.0	ns	29.8	***	25.5	***	12.7
**Smoking status, %**			ns		ns		ns		ns	
Daily smoker	95.7	94.4		93.4		95.8		94.4		95.4
Weekly/monthly smoker	4.3	5.6		6.6		4.2		5.6		4.6
**CPD, mean (SE)**	16.4 (0.2)	17.3 (0.4)	*	16.9 (0.8)	ns	18.2 (0.8)	*	17.2 (0.4)	ns	16.6 (0.2)
**Time to first cigarette after waking, %**			ns		ns		ns		ns	
>30 minutes	34.1	31.8		31.2		31.2		32.2		33.4
6–30 minutes	42.3	43.8		37.6		41.0		43.0		42.7
≤5 minutes	23.6	24.4		31.2		27.8		24.8		23.9
**Ever e-cigarette use, %**	19.6	24.6	**	18.6	ns	20.4	ns	26.9	***	20.7
**Current e-cigarette use, %**	3.0	3.9	ns	1.6	ns	3.0	ns	4.2	ns	3.2

Note: p-values refer to tests conducted comparing individuals with PAD, TDD, TDA, or PSD compared to individuals with No PAD. ns: non-significant.

* p<0.05

** p<0.01

*** p<0.001.

PAD: Probable Anxiety or Depression = TDA or TDD or PSD. CPD: cigarettes per day.

### Smoking behaviors

Respondents with PAD smoked an average of 17.3 (SD= ±0.4) cigarettes per day, which was significantly higher compared to smokers without PAD. Smokers with TDA also smoked significantly more than respondents without PAD. The majority (68.2%) of smokers with PAD reported smoking within 30 minutes of waking up in the morning, with no significant differences documented among respondents with and without PAD (Table 2).

### E-cigarette use

Ever use of an e-cigarette was more frequently reported by smokers with PAD and those with a PSD compared to those without PAD (Table 3). Current e-cigarette use did not differ based on presence of PAD.

**Table 3:** Past quit attempts, quitting methods, self-efficacy and readiness to quit by presence of probable anxiety or depression, pooled sample

*Variable*	*No PAD (n=4575)*	*Probable Anxiety or Depression (PAD) Definitions*							

		*PAD (n=1284)*		*Current treatment/diagnosis for depression (TDD) (n=224)*		*Current treatment/diagnosis for anxiety (TDA) (n=303)*		*Positive screen for depression (PSD) (n=1115)*	

	*%*	*%*	*p*	*%*	*p*	*%*	*p*	*%*	*p*
**Ever tried to quit smoking, yes**	51.4	62.2	***	52.1	ns	54.2	ns	65.1	***
**Made attempts to quit smoking in the last 12 months, yes**	15.4	24.7	***	19.0	ns	18.1	ns	26.2	***
**Number of quit attempts in the last 12 months**			***		ns		ns		***
No attempt	85.1	76.4		82.8		84.0		74.5	
1 attempt	9.1	10.9		7.3		7.4		11.7	
2 or more attempts	5.7	12.6		9.9		8.6		13.8	
**How long ago did your most recent quit attempt start?^1^**			ns		*		ns		ns
Less than 1 month ago	14.3	16.0		29.4		20.5		15.3	
1–3 months ago	31.0	30.2		30.1		33.7		31.0	
4–6 months ago	26.6	30.8		24.1		29.2		30.1	
7 months or more ago	28.1	23.0		16.5		16.6		23.6	
**Duration of most recent quit attempt^1^**			ns		ns		ns		ns
Less than 1 day	11.8	10.8		12.6		12.2		10.4	
1–6 days	32.4	34.7		36.8		37.7		36.6	
1–4 weeks	31.1	31.6		33.4		35.4		30.3	
1–6 months	20.6	20.8		16.1		13.2		20.6	
7–12 months	4.1	2.2		1.1		1.5		2.1	
**Use of support during most recent quit attempt**			ns		ns		**		
No support	71.9	70.5		58.9		51.7		72.3	ns
Any support	28.1	29.5		41.1		48.3		27.7	
**Quit methods used during most recent quit attempt^2^**									
Nicotine replacement therapy	10.1	11.3	ns	25.0	**	32.8	***	8.6	ns
Pharmacotherapy (varenicline, bupropion, cytisine)	7.9	15.4	ns	24.1	**	11.6	ns	15.2	ns
Local quit services	0.9	1.8	*	6.9	**	9.9	***	0.4	ns
Face to face advice from health care professional	4.3	8.5	*	15.0	**	17.5	***	7.0	ns
Telephone/quitline services	0.1	2.4	***	9.0	***	6.0	***	1.9	***
E-cigarette or vaping device	14.3	13.7	ns	13.4	ns	22.5	ns	12.9	ns
**If you decided to quit, how sure are you that you would succeed at quitting smoking**			ns		ns		ns		ns
Not at all sure	34.6	34.1		34.9		36.5		35.4	
Slightly/moderately sure	51.8	50.2		49.9		50.7		48.7	
Very/extremely sure	13.6	15.7		15.3		12.8		15.8	
**How difficult would it be for you to quit smoking if you wanted to**			ns		ns		ns		ns
Not at all/slightly difficult	25.8	24.9		27.3		24.7		23.9	
Moderately difficult	27.1	24.7		22.9		22.7		24.8	
Very/extremely difficult	47.1	50.4		49.8		52.6		51.3	
**Readiness to quit**			***		**		**		***
Within the next month	3.4	5.7		4.6		2.5		6.2	
Within the next 6 months	7.2	12.2		13.2		12.3		11.8	
Sometime in the future, >6 months	29.8	38.0		36.5		37.7		38.6	
Not planning to quit	59.7	44.2		45.7		47.5		43.4	
**How much do you want to quit smoking^2^**			***		ns		ns		***
A little	22.3	15.9		17.3		15.0		15.8	
Somewhat	47.4	38.2		48.2		45.8		35.0	
A lot	30.4	45.8		34.4		39.2		49.2	

Note: p-values refer to tests conducted comparing individuals with PAD, TDD, TDA, or PSD compared to individuals with No PAD. ns: non-significant.

* p<0.05

** p<0.01

*** p<0.001.

PAD: Probable Anxiety or Depression = TDA or TDD or PSD.

1 Based on subsample of those who had tried to quit smoking in the last 12 months (N=1056).

2 Based on subsample of those who plan to quit (N=2514).

### Past quit attempts and cessation assistance used

Having ever made a quit attempt was reported more frequently among respondents with PAD (62.2% vs 51.4%; p<0.001), as well as with a PSD (65.1% vs 51.4%; p<0.001), compared to those without PAD (Table 3). Having made an attempt within the last 12 months was also significantly higher among participants with PAD and with a PSD compared to those without PAD (24.7% and 26.2% vs 15.4%; p<0.001). Making two or more quit attempts within the past 12 months was significantly greater among those with reported PAD and with a PSD compared to those without PAD [(12.6% vs 5.7%; p<0.001) and (13.8% vs 5.7%; p<0.001)].

Among those who had made a quit attempt within the last 12 months, the vast majority tried to quit without support, with no differences documented between smokers with and without PAD. Smokers with TDA were significantly more likely to have tried to quit with support. Among those respondents with PAD who used support, the most frequently reported quit method was prescription-based quit smoking pharmacotherapy (15.4%) followed by e-cigarettes (13.7%) and NRT (11.3%). NRT and pharmacotherapy were significantly more likely to have been used as part of past quit attempts by respondents with a TDD. NRT was also more frequently used by individuals with TDA. Person-to-person behavioral support (i.e. local quit services, face-to-face advice from a doctor or other health care professional, telephone or quitline services) was reported significantly more frequently among respondents with PAD, TDA, TDD compared to those without these conditions.

### Readiness to quit and self-efficacy

Respondents with PAD and the sub-categories of PAD were more likely to report readiness to quit smoking in the future compared to those without PAD (Table 3). Statistically significantly more smokers with PAD or PSD reported that they wanted to quit smoking ‘a lot’ compared to those without PAD. No significant differences in respondent’s self-efficacy with quitting were found based on diagnosis of PAD in the unadjusted analysis.

### Multivariable analysis

Results of the multivariable logistic regression analysis adjusting for sex, age, education, country, ability to pay bills on time, and cigarettes smoked per day, showed that respondents with PAD were more likely to have ever made a past attempt to quit smoking, to have made a quit attempt in the last 12 months, and to report lower self-efficacy compared to respondents without PAD (Table 4). Individuals with PAD were also more likely to report having received advice to quit from a doctor or health professional and having used quitline support as part of their last quit attempt (Table 4). Readiness to quit smoking was no longer significantly different among respondents with and without PAD after adjusting for cigarettes smoked per day.

**Table 4:** Logistic regression analysis examining correlates of probable anxiety or depression (PAD)

*Dependent variables (Ref. group=No PAD)*	*AOR (95% CI)^1^*	*p*
Time to first cigarette in morning (within 30 minutes)^2^	1.00 (0.81–1.23)	0.961
Ever tried to quit smoking^3^	1.48 (1.25–1.74)	<0.001
Attempt to quit smoking in the last 12 months^3^	1.75 (1.45–2.11)	<0.001
Use of any support during last quit attempt^3^	1.05 (0.70–1.57)	0.822
Advice from doctor/health care professional as part of last quit attempt^3^	2.38 (1.21–4.71)	0.013
Used telephone/quitline services as part of last quit attempt^3^	19.04 (2.51–144.55)	0.005
Intend to quit smoking in the next six months^4^	0.74 (0.60–0.91)	0.005
Self-efficacy^5^	1.83 (1.44–2.32)	<0.001
Perceived difficulty with quitting^6^	1.09 (0.91–1.31)	0.329
Wants to quit smoking ‘somewhat’ or ‘a lot’^7^	1.39 (1.05–1.85)	0.023

Ref. group=No PAD. AOR: adjusted odds ratio, CI: confidence interval.

1 Adjusted for sex, age, education, country, ability to pay bills on time, cigarettes per day.

2 ‘How soon after waking do you usually have your first smoke?’; Dichotomized response: 1 = ≤5 minutes or 6–30 minutes, 0 ≥30 minutes.

3 Response: 1=yes, 0=no.

4 ‘Are you planning to quit smoking?’; Dichotomized response: 1 = ‘within the next month’ or ‘within the next 6 months’, 0 = ‘sometime in the future, > 6 months’ or ‘not planning to quit’.

5 ‘If you decided to quit, how sure are you that you would succeed at quitting smoking?’; Dichotomized response: 1 = ‘not at all sure’ or ‘slightly/moderately sure’, 0 = ‘very/extremely sure’.

6 ‘How difficult would it be for you to quit smoking if you wanted to?’; Dichotomized response: 1 = ‘extremely difficult’, 0 = ‘moderately difficult’ or ‘not at all/slightly difficult’.

7 Dichotomized response: 1= ‘somewhat’ or ‘a lot’, 0 = ‘a little’.

## DISCUSSION

Tobacco control efforts have escalated in Europe with the entry into force of the EU TPD 2014/40/EU in May 2016 and the ratification of the WHO FCTC, both aimed at reducing the morbidity and mortality of tobacco use^22^. With elevated smoking prevalence and smoking-attributed deaths among individuals with mental illness^1,2^, in order for these legislative efforts to maximize their impact, it is critical to ensure that the unique barriers facing this population of smokers in achieving cessation are understood and subsequently addressed with the implementation of the EU TPD. As such, the objective of the current study was to provide insight into the population of tobacco users with probable anxiety or depression (PAD) in Europe in order to inform tailored intervention strategies and as a baseline for monitoring how the EU TPD will impact on this population over time.

Our study found that 21% of smokers sampled from six EU MS had a diagnosis, treatment or positive screen for anxiety or depression, with rates of PAD varying between EU MS. Smokers with PAD were more likely to report greater daily cigarette consumption, a failed past quit attempt in their lifetime as well as in the previous 12 months, and to report readiness to quit smoking in the future compared to smokers sampled without PAD, consistent with previous research studies^29-31^. Respondents with PAD also reported increased economic challenges (i.e. lower household income and/or ability to pay bills on time) than the general population of tobacco users. Prevalence of anxiety and depression in our study should be considered against a prevalence of 25% reported in Europe^32^. It should be noted that these data are based on a broader sample of EU countries relative to the present study and refer to both smokers and nonsmokers. Differences noted may also be due to differing definitions of anxiety and depression disorders between population surveys^7-9,21^.

Consistent with previous reports, we found prevalence of depression was higher among women^30,31^ and those of lower socioeconomic status^32,33^. Importantly, while less than 5% had a current diagnosis or were receiving treatment for anxiety or depression, 18.3% of the sample screened positive for major depressive disorder. This suggests a possible treatment gap among this population of smokers with PAD. This is consistent with previous reports that suggest that half of major depressions among Europeans are untreated, which among smokers may particularly undermine cessation efforts^32,33^.

Our data are consistent with previous findings, which have found smokers with mental health illness are as motivated to quit as the general population of smokers; with a greater proportion of smokers with PAD in our study reporting an interest in quitting smoking sometime in the future compared to smokers without PAD^10,15,34^. It is important to highlight that, although there were no statistically significant differences between smokers with and without PAD regarding interest in quitting, the majority of smokers with PAD indicated it would be very or extremely difficult to quit smoking. Importantly after adjusting for socioeconomic factors and cigarettes smoked per day, respondents with PAD had significantly lower confidence in their ability to quit smoking successfully. Given that a large proportion of patients with PAD attempted to quit multiple times unsuccessfully, this may have reduced their confidence in their ability to quit in the future as well as their intentions for quitting.

Available data suggests that rates of tobacco use are higher, and quit rates lower, among patients with mental illness, including those with anxiety and depression, compared to the general population of smokers, making this group of smokers an important sub-population for future intervention and treatment^3,5,7^. Smokers with PAD reported significantly more frequent failed quit attempts relative to those without PAD. These relapses may be indicative of poor coping strategies for addressing withdrawal, cravings, negative affect and mood^1,5^. We found significantly higher levels of daily nicotine consumption among smokers with PAD relative to those without (17.3 vs 16.4 cigarettes/day), which is consistent with those reported for North American and UK populations^3,7,19^. Sampled rates of daily cigarette consumption among smokers with PAD were found to be greater than the EU average (17.3 vs 14.1, respectively) and would be considered high^25^. Likewise, the vast majority of tobacco users reported high rates of nicotine dependence, with almost 70% of the sample reporting time to first cigarette in the morning was within 30 minutes of waking up; however, this was not statistically different when compared to those without PAD.

Thirty-three per cent of smokers with PAD reported past use of an e-cigarette and were more likely to report past use of e-cigarettes than those without PAD. These findings are consistent with earlier research, which found that individuals with mental illness were more likely to have experimented and used e-cigarettes, compared to the general population of tobacco users^35,36^. Possible explanations for experimentation with e-cigarettes have been discussed by others and include greater optimism among smokers with mental illness about the health benefits of e-cigarettes compared to regular cigarette use and thinking less about how e-cigarettes might potentially harm their health^35,37^. It may also be the case that individuals who choose to use e-cigarettes are interested in reducing the harms associated with their continued smoking. However, in contrast to previous research current e-cigarette use was not found to be greater among patients with PAD. These findings have implications in terms of preference for quitting, quitting methods and cessation outcomes among patients with mental illness.

### Implications to future research and practice

Perhaps the most important data to be highlighted are the documented rates of untreated depression among this population of smokers, the frequency of failed quit attempts, and the low prevalence of use of evidence-based smoking cessation methods as part of past quit attempts, despite high levels of nicotine dependence. Specifically, while quitting and use of quit methods of past quit attempts were significantly greater among smokers with PAD, the overall use of pharmacological and non-pharmacological aids remained extremely low with 1 in 10 smokers with PAD reporting use of a cessation method as part of their last quit attempt. These data are consistent with previous reports and emphasize the need to provide appropriate treatment support to this population of tobacco users^10,11^. Specifically, behavioral counselling that includes a psychosocial mood management component to a standard smoking cessation intervention has been shown to increase long-term cessation rates in smokers with both current and past depression, compared with standard interventions^5^. Our study found rates of pharmacotherapy to be higher among patients with PAD, however they were still only used by 10–15% of tobacco users who made a quit attempt. Due to past concerns regarding potential neuropsychiatric adverse effects of quit-smoking pharmacotherapy there may have been a reluctance among both patients and health care professionals to use these pharmacotherapies in this population of patients with mental illness. However, Anthenelli et al.^16^ in a large multinational investigation found these medications to be safe and superior to placebos in achieving long-term abstinence among patients with mental illness, with no significant adverse events. Given that both pharmacological and non-pharmacological quit smoking aids have been safe, acceptable and effective for people with and without mental illness, it is important that their use be promoted among smokers with anxiety, depression and other forms of mental illness alongside behavioral counseling^4,5,16^. Our findings support the need for interventions targeting health care professionals in providing smoking cessation assistance to this population of smokers^20^.

Importantly, countries such as Australia, Canada, the USA and UK, which have made large investments in tobacco control efforts over an extended time period, report much lower prevalence of tobacco use (AU:17%, CA:17%, UK:17%, USA:15.5%)^38-40^, however the proportion of smokers with anxiety and/ or depression in Australia (27%), the USA (36.1%), and UK (33%) is higher compared to the six EU countries included in this study^3,7-10,19,21^. Evidence from these countries suggests that as the total number of smokers in a country decreases over time, as a result of investments in tobacco control, the proportion of smokers with mental illness increases^3,7-9,19-21^. This trend appears to be due to the fact that people with mental illness find it harder to quit, and as such are less responsive to public health initiatives that are not targeted to their specific needs. Over time, as those smokers without mental illness quit, an ever-increasing proportion of remaining smokers have a mental illness. To combat this trend, it will be important for public health interventions to target the specific needs of smokers with mental health illness.

### Strengths and limitations

The strengths of this study include a large sample of about 6000 participants from six EU countries and examination of an extensive number of variables and use of validated assessment tools. To our knowledge, this is one of the few studies to describe the behavior of smokers with PAD in European countries other than the United Kingdom. Our study also has several limitations. First, the cross-sectional design of the study prevents us from identifying causal inferences as opposed to associations. Second, our study relies on self-report and should not, as such, be inferred as a medically confirmed diagnosis of PAD. The self-report of socially undesirable behaviors and conditions, such as tobacco smoking or mental health status, may be subject to reporting bias. Likewise, socially desirable behaviors (e.g. lower numbers of cigarettes smoked per day, intentions of quitting) may be over-reported. Our study was limited to the collection of data assessing probable anxiety or depression; we did not as such examine the presence of other mental disorders (i.e. panic disorders, bipolar disorder, lighter forms of depression or mood disorders). It is also possible that some respondents may have other mental disorders in addition to anxiety and depression with specific characteristics of these sub-groups requiring further examination. This study involved a community-based sample, which would exclude those living in mental health institutions or facilities etc., who are more likely to be receiving treatment for more severe mental health illnesses. In the present study, all participants were current smokers and so precluded making comparisons between successful and unsuccessful quit attempts, assessing predictors of cessation outcomes, or evaluating the cessation rates.

## CONCLUSIONS

In this multi-country European study, we found that around one in four smokers had probable anxiety or depression. Smokers with PAD had a greater interest in quitting but more frequent failed quit attempts than smokers without PAD. Among those with PAD, we observed high rates of nicotine dependence, low confidence in their ability to quit in the future, infrequent use of cessation methods, and low socioeconomic status, which may make quitting even more difficult. Future intervention should look at increasing quitting confidence and use of evidence-based cessation methods among tobacco users with PAD, as well as targeting health care professionals to strengthen cessation assistance provided to this population. Understanding the characteristics and past quitting experiences of tobacco users with anxiety or depression is essential for improving treatment strategies.

## CONFLICTS OF INTEREST

The authors declare that they have no competing interests, financial or otherwise, related to the current work. P. Katsaounou reports grants and personal fees from GSK, Astra Zeneca, BI, Chiesi, Pfizer, Menarini, outside the submitted work. A. Herbeć reports grants from Pfizer, outside the submitted work. C. I. Vardavas reports that he is the Strategic Development Editor of TID and that there are no conflicts of interest with this current work. The rest of the authors have also completed and submitted an ICMJE form for disclosure of potential conflicts of interest.
