# Corrigendum: Patterns of tobacco use, quit attempts, readiness to quit and self-efficacy among smokers with anxiety or depression: Findings among six countries of the EUREST-PLUS ITC Europe Surveys

**DOI:** 10.18332/tid/114415

**Published:** 2019-11-22

**Authors:** Ioanna Petroulia, Christina N. Kyriakos, Sophia Papadakis, Chara Tzavara, Filippos T. Filippidis, Charis Girvalaki, Theodosia Peleki, Paraskevi Katsaounou, Ann McNeill, Ute Mons, Esteve Fernández, Tibor Demjén, Antigona C. Trofor, Aleksandra Herbeć, Witold A. Zatoński, Yannis Tountas, Geoffrey T. Fong, Constantine I. Vardavas, on behalf of the EUREST-PLUS consortium

**Affiliations:** 1 National and Kapodistrian University of Athens (UoA), Athens, Greece; 2 European Network on Smoking and Tobacco Prevention (ENSP), Brussels, Belgium; 3 University of Crete (UoC), Heraklion, Greece; 4 Division of Prevention and Rehabilitation, University of Ottawa Heart Institute, Ottawa, Canada; 5 Department of Primary Care and Public Health, School of Public Health, Imperial College London, London, United Kingdom; 6 King’s College London (KCL), London, United Kingdom; 7 Cancer Prevention Unit and WHO Collaborating Centre for Tobacco Control, German Cancer Research Center (DKFZ), Heidelberg, Germany; 8 Tobacco Control Unit, Catalan Institute of Oncology (ICO), and Cancer Control and Prevention Group, Bellvitge Biomedical Research Institute (IDIBELL), Catalonia, Spain; 9 School of Medicine and Health Sciences, Universitat de Barcelona, Catalonia, Spain; 10 Smoking or Health Hungarian Foundation (SHHF), Budapest, Hungary; 11 University of Medicine and Pharmacy ‘Grigore T. Popa’ Iasi, Iasi, Romania; 12 Aer Pur Romania, Bucharest, Romania; 13 Health Promotion Foundation (HPF), Warsaw, Poland; 14 European Observatory of Health Inequalities, President Stanisław Wojciechowski State University of Applied Sciences, Kalisz, Poland; 15 Department of Psychology, University of Waterloo (UW), Waterloo, Canada; 16 School of Public Health and Health Systems, University of Waterloo (UW), Waterloo, Canada; 17 Ontario Institute for Cancer Research, Toronto, Canada

**Keywords:** depression, smoking cessation, anxiety, mental health, Europe

Tobacco Induced Diseases, Volume 16, Special Issue: Supplement 2, Pages 1–13. Publish date: 5 February 2019, DOI: https://doi.org/10.18332/tid/98965

On the 9th page, 1st paragraph of the article, the original phrase ‘Prevalence of anxiety and depression in our study should be considered against a prevalence of 25% reported in Europe^32^’, should be corrected to: ‘Prevalence of anxiety and depression in our study should be considered against a prevalence of anxiety and depression of 4.3% and 5.1%, respectively, reported in Europe in 2015^32^’.

